# Differences in daily life executive functioning between people with autism and people with schizophrenia

**DOI:** 10.1007/s10803-022-05547-6

**Published:** 2022-04-20

**Authors:** Jo A. Yon-Hernández, Dominika Z. Wojcik, Laura García-García, Manuel A. Franco-Martín, Ricardo Canal-Bedia

**Affiliations:** 1 Instituto Universitario de Integración en la Comunidad (INICO), Universidad de Salamanca, Avda. de la Merced, 109-131, 37005 Salamanca, Spain; 2 Zamora Hospital (Complejo Asistencial de Zamora), Zamora, Spain

**Keywords:** Executive functions, DEX-Sp, Autism spectrum disorder, Schizophrenia spectrum disorders, Adaptive behavior

## Abstract

This is a comparative analysis of everyday executive functioning between individuals with Autism Spectrum Disorder (ASD), Schizophrenia Spectrum Disorders (SSD) and controls using Dysexecutive Questionnaire-Spanish (DEX-Sp), to identify patterns of difficulties. Also we assessed the relationship between EF and adaptive behavior as measured by the Vineland Adaptive Behavioral Scale-II. Common areas of everyday executive functions were established as problematic in individuals with ASD and SSD related to Disinhibition and Apathy, while Disorganization and Impulsivity was gravely affected in ASD group only. The degree of Dysexecutive Syndrome was predictive of adaptive behavior in ASD group only. These suggest that DEX-Sp could be a useful tool in differentiating areas of strength and weaknesses in clinical groups such as ASD and SDD.

Autism spectrum disorder (ASD) and schizophrenia spectrum disorders (SSD) are two conditions with high co-occurrence. Lugo-Marín et al., (2018), for example, showed that the prevalence of SSD in adults with ASD is near 6%. Autistic traits can be frequently found in individuals with SSD and some symptoms of SSD can also be present in ASD (Spek & Wouters, 2010; Wouters & Spek, 2011). This overlap of symptoms is seen in limited social responsiveness, in the social withdrawal as well as in the lack of interest in everyday social interactions (Spek & Wouters, 2010).

Recent studies indicate that both disorders share genetic factors, such as recurrent copy number variations (CNV) in chromosomal regions (Burbach & van der Zwaag, 2009; Cheung et al., 2010). Also, neuroimaging studies show that the two disorders share abnormal brain development. For example, an alteration of the hippocampus, associated with diminished performance on behavioral, language and emotional response measures (Cheung et al., 2010) was found in both conditions. Both people with autism and with schizophrenia showed different volume of cerebellum, hippocampus and amygdala when compared to typically-developed individuals (Stone & Iguchi, 2011). However, little is known about how these similarities in genetic and brain profiles translate to everyday difficulties that people with SSD and ASD experience. Our main goal, therefore, is to examine these individuals’ performance on day-to-day life activities such as planning, organizing or carrying out tasks and see how this performance may affect their adaptive behavior. We specifically want to see how the two conditions differ on everyday executive function tasks because identifying differences may be helpful for differential diagnosis of the two disorders as well as for designing appropriate treatment.

Autism spectrum disorder and SSD have been characterized by the Diagnostic and Statistical Manual of Mental Disorders (DSM-5) under conditions with impairments in skills related to daily life functioning (American Psychiatric Association, 2013). Daily living skills are often referred to as adaptive functioning that encompasses basic activities such as personal hygiene, dressing properly, toileting, feeding, as well as more complex activities such as meal preparation, independent mobility, household chores, health and medication management. All of these skills are important for meeting the environmental demands in daily life and for maintaining a reasonable level of health and safety. Deficits in adaptive behavior are highly prevalent in ASD and they contribute to the overall poor outcomes in adults with ASD (Duncan & Bishop, 2015). A recent study in 50 to 80-year-old adults with ASD reported important difficulties in everyday life activities, especially in organizing and executing plans (Davids et al., 2016). Moreover, adults with autism remain dependent on their parents, care providers or relatives well into their adulthood because of the difficulties they face in their everyday life (Davids et al., 2016; Geurts & Vissers, 2012). Likewise, people with SSD have adaptive difficulties. For example, Leifker et al., (2009) examined the extent to which symptomatology as well as cognitive and social impairments, affect the ability of individuals with SSD to function in daily life. Specifically, they reported difficulties related to personal care (e.g., eating, toileting etc.), participation in community activities (e.g., shopping, paying bills, using public spaces, etc.) and work skills (e.g., independent self-sufficiency or punctuality).

Poor adaptive behavior in both conditions has been attributed to deficits in intellectual functioning (as measured by Intellectual Quotient - IQ). However, recent studies in ASD indicated that individuals without intellectual disabilities show poorer adaptive behavior to what would be expected for their cognitive ability level (Kanne et al., 2011; Kraper et al., 2017; McQuaid et al., 2021; Nyrenius & Billstedt, 2020; Pathak et al., 2019; Zukerman et al., 2021). What is more, problems in adaptive behavior in ASD individuals with average/high IQ persist throughout the development (McQuaid et al., 2021; Nyrenius & Billstedt, 2020; Pathak et al., 2019; Zukerman et al., 2021), suggesting that the low IQ does not suffice to explain the limitations in adaptive functioning.

On the other hand, the limitations often seen in daily living skills in schizophrenia have been associated with their cognitive deterioration and low IQ (Harvey & Strassnig 2012). Indeed, some researchers agree that intellectual functioning could explain poor adaptive behavior in SSD (Fiksinski et al., 2019). For example, long-term memory (Bhattacharya 2015), selective and sustained attention as well as executive functions are all known to affect adaptive behavior (Godbout et al., 2007; Greenwood, 2005; Rempfer et al., 2003; Semkovska et al., 2004) and have also all been found to be impaired in SSD. Given the role of intellectual ability in adaptive behavior, our study will control for IQ, to see how other variables, specifically Executive Functions (EF), contribute to adaptive behavior deficits in both conditions.

There are several approaches that attempt to explain what factors contribute to adaptive behaviors, with the explanation of the EFs being one of the most prominent one. Executive functions refer to a set of higher-order cognitive processes (Demetriou et al., 2019), such as response initiation, selection and strategy formation, flexibility, inhibition of prepotent responses (Bramham et al., 2009; Johnston et al., 2019) and future planning (Demetriou et al., 2019). These functions are necessary to respond effectively to environmental demands as they cover a broad range of domains that enable us to regulate goal-directed behavior (Vogan et al., 2018), plan, or flexibly change strategies in immediate contexts (Wallace et al., 2016).

A vast amount of research in ASD and SSD has shown that both disorders are characterized by impaired executive functioning (see Demetriou et al., 2018; Vogan et al., 2018). Adults with ASD, for example, showed poor performance on neuropsychological EF tasks (e.g., Zoo Map and Key Search) such as planning, generativity of novel solutions and flexibility (Wallace et al., 2016). Although research into inhibitory control in ASD, as measured by standardized neuropsychological tools, has produced mixed results, there is evidence suggesting deficits. For instance, limitations and difficulties in inhibiting prepotent responses and suppressing distractors have been confirmed (for a review see Hlavatá et al., 2018; Zhang et al., 2020). Also, automatic inhibition of visual, auditory and verbal responses was found to be impaired in both ASD and SSD (Shi et al., 2020).

A systematic review by Bhattacharya (2015) into EF in SSD found that monitoring, working memory, planning, switching and inhibitory control were among the most frequently affected EFs. Also, mental flexibility and the ability for abstraction have been linked to EF impairments in this disorder (Clark et al., 2010). Furthermore, in a series of verbal fluency tasks, Iampietro et al.(2012) found a perseverative response pattern as well as an impairment in response monitoring, which suggests that individuals with SSD have difficulties to switch or select an appropriate response on demand.

Despite the clear overlap in terms of the impoverished performance on EF tasks in ASD and SSD (Clark et al., 2010), few studies targeting task-based EFs have been conducted comparing the two clinical populations. Barlati et al., (2019), analyzed the cognitive profiles of patients with ASD and SSD using the raw scores for each of the WAIS-R (Wechsler, 1981) subtests (Information, Digit Span, Vocabulary, Arithmetic, Comprehension, Similarities, Picture Completion, Story-arranging, Block Design, Object Construction, and Coding) demonstrating that individuals with schizophrenia and autism have difficulties in working memory and processing speed tasks. Using the same assessment test, another study also found difficulties in working memory and processing speed tasks in both ASD and SSD participants (Marinopoulou et al., 2016). Yet another study that used the Dysexecutive Syndrome Questionnaire, found college students with ASD with schizotypal traits to have poorer performance in tasks that measure planning and flexibility compared to their typical developed classmates (Shi et al., 2017; Wilson et al., 1996). Also Stone & Iguchi (2011) observed that perceiving, organizing and integrating information from the environment is problematic in both ASD and SSD, and that both groups fail to inhibit irrelevant information.

All the above-mentioned EFs are important to generate goal-directed behaviors and subsequently to learn and generate adaptive behaviors in the service of handling everyday life situations. Studies examining the role of EFs in adaptive behavior in children and adolescents with ASD indicate fewer difficulties when tasks are structured and the environment has no distractions, in contrast to real-life situations where EFs are necessary to interpret, process and perform adaptive behaviors (Gardiner & Iarocci, 2018). Similar outcomes can be seen in adults with schizophrenia. Velligan et al., (2000) conducted a randomized controlled trial using cognitive adaptation training, targeting adaptive functioning in schizophrenia. They found improvements in the participants who received personalized prompts and cues (e.g., using checklists, placing signs, summarizing steps, using labels etc.) in contrast to the participants who only received clinical orientations on what activities they needed to improve in their daily lives.

Although ASD is a lifelong disorder, and difficulties in EF and adaptive behavior are known to continue into adulthood, little is known about the relationship between everyday EF impairments among adults with ASD and their role in adaptive behavior. This lack of research is somewhat surprising, given that EF affects multiple aspects of an individual’s performance in day-to-day activities. In the case of SSD, EF impairments can be associated with difficulties coping with roles in community settings, such as a reduction in social abilities, an unsuccessful vocational path (Bhattacharya, 2015), and with a poor quality of life (Clark et al., 2010). Meanwhile, ASD problems in EF can be linked to adjusting to new settings and coping with the roles expected from others as part of their transition into adulthood (Matthews et al., 2015). These findings suggest that individuals with ASD and SSD will need guidance or intervention to perform everyday tasks or to respond adequately to environmental demands (Fiksinski et al., 2019; Geurts & Vissers, 2012).

While research has focused on determining similarities between ASD and SSD, such as social functioning, emotion regulation, or cognition (Barlati et al., 2019; Marinopoulou et al., 2016), we believe that studying differences in each condition can also be clinically significant. Comparative studies, like the present one, can help us to better understand the specificity of problems in everyday life that individuals with each condition experience and further inform us what aspects of EF should be targeted in each condition. The long-term objective, here, is to advance our knowledge on adequate clinical supervision tailored to specific needs that each group might manifest. For example, the intensity and duration of intervention suitable for each ASD and SSD could be derived from studies that look at severity of impairments and adaptive behaviors. In the case of adults with autism, there are not as many intervention programs and specific therapeutic strategies as there are for SSD. Therefore, the most valuable aspect of the comparison study between autism and schizophrenia is that it could provide results showing that, although both disorders may have equally impaired performance in different domains of adaptive functioning, the cognitive dysfunctions may not necessarily share the same underlying mechanisms (Zhang et al., 2015). Consequently, any findings in this direction would help clinicians to develop intervention strategies and approaches that are more personalized and tailored to specific difficulties that individuals with each disorder have.

Therefore, our first objective was to conduct a comparative analysis of cognitive impairments in everyday executive functioning between ASD and SSD samples compared to controls. To do that, we used the Dysexecutive Questionnaire-Spanish (DEX-Sp) (Pedrero-Pérez et al., 2011, 2015), a tool that typically detects EF difficulties in clinical populations that are known to have EF problems such as the Dysexecutive Syndrome – DS (Yang et al., 2018; Shi et al., 2017), found a stronger relationship between impaired EFs with autistic traits than EF and schizotypal traits. We expected to find a similar pattern of results, whereby participants with ASD would show greater deficits than participants with SSD. We strengthened the methodology of the previous study by using clinical groups with a well-stablished diagnosis, as opposed to groups with autistic or schizotypal traits. Also, the novelty of our study was to control for factors such as IQ when looking at performance on real-world executive functioning in each clinical group.

Secondly, we explored the relationship between everyday executive functioning using the DEX-Sp and adaptive behavior, using the Vineland Adaptive Behavioral Scale-II (VABS-II). The latter test is commonly used to assess adaptive behaviors in both ASD and SSD groups. Because previous research has also found that poor performance on EF was associated with poor outcomes in day-to-day settings in both groups, we hypothesized that impairments in everyday executive functioning (measured with DEX-Sp) should predict adaptive behavior in daily living skills in ASD and SSD (measured with VABS-II).

Research in this field is highly relevant due to a limited amount of empirical evidence examining the usefulness of the above-mentioned assessment tools in clinical populations of ASD and SSD (Barlati et al., 2019). Reliable measurements are necessary to explore everyday EFs to detect a pattern of strengths and weaknesses in each disorder. To our knowledge, this is the first time DEX-Sp is used to compare adults with these disorders on everyday EF as well as to examine EF role in adaptive behaviors.

## Methods

### Participants

A total of 89 individuals took part in this study, including participants with ASD, SSD and the Control group. All groups were an opportunity sample. To assess possible autistic traits in all three groups the Autism Spectrum Quotient Short Form Spanish version (AQ-S) (Lugo-Marín et al., 2019) was administered. The cut-off point for autistic traits is > 63 (see Table 1). Below we describe each group’s characteristics:

### ASD group

Thirty-five individuals with ASD took part in the study (21 males, 14 females; age range 16–54 years old). Participants were diagnosed by a clinical and diagnostic team using the Autism Diagnostic Observation Schedule-ADOS-2, Module 4 (Lord et al., 2015). Due to time constraints, we could not confirm the diagnosis of four participants with the ADOS-2. However, given that these participants had a previous formal diagnosis by certified clinicians, we believe no methodological implications arise from this lack of confirmatory assessment. All the participants in this group met criteria for autistic traits as measured by the AQ-S (see Table 1).

### SSD Group

Twenty-two individuals with SSD participated in the study (18 males, 4 females; age range 21–62 years old). They were recruited randomly from the Psychiatry and Mental Health Service of the Hospital. The diagnoses were established prior to the present study and all the participants met DSM-5 diagnostic criteria for SSDs. Exclusion criterion for this group was the presence of acute psychotic symptoms at the time of the evaluation which was measured using the Positive and Negative Syndrome Scale (PANSS) Spanish version (Kay et al., 1987; Peralta & Cuesta, 1994). Higher scores denoted greater psychotic symptomatology. Results on the PANSS reflected no acute psychotic symptoms at the time of the study (see Table 1). To take part in the study the participants had to show no recent or previous history of substance abuse (e.g., alcohol, cannabis, hallucinogens or opioids) for a period of more than 5 years. Finally, antipsychotic medication doses in all cases were administered under the guidelines of the Agencia Española de Medicamentos y Productos Sanitarios. To assess autism co-occurrence, the ADOS-2 was also administered to this group, where three participants were not available for testing. Five participants in the ADOS-2 scored above the cut-off point for Autistic Spectrum Disorder and 11 participants scored above the cut-off point for the AQ-S.

### Control group

Thirty-two individuals were recruited for this group (20 males, 12 females; age range 18–63 years old). Participants from the control group were university students from different faculties and people from the general public. The exclusion criterion for this group was to score below the cut-off point for the AQ-S. None of the participants were excluded.

A pre-study clinical questionnaire about co-occurring disorders, neurological diseases, or brain damage was obtained from the participants. One participant in the ASD group reported having epilepsy and 7 participants from the ASD group reported being under some type of medication (see Table 1).

**Table 1:** Participants’ demographic characteristics by groups.

			Group			Independent-Samples Kruskal-Wallis Test			
		Mean (SD)							
			Control (n = 32)	ASD (n = 35)	SSD (n = 22)	*H*	df	*p*	Differences ^a b^
Age			29.91 (10.51)	29.83 (11.54)	46.36 (11.10)	24.10	2	.001	SSD > (ASD = Control)
WAIS-Full Scale IQ			115.56 (15.21)	103.74 (22.86)	93.50 (18.70)	14.60	2	.001	SSD < (ASD = Control)
WAIS-Verbal IQ			128.69 (15.17)	116.34 (23.13)	111.45 (21.88)	9.12	2	.010	SSD < (ASD = Control)
WAIS-Performance IQ			106.41 (17.12)	98.69 (23.12)	89.18 (19.71)	9.51	2	.009	SSD < (ASD = Control)
AQ-S			51.03 (7.66)	74.91 (11.94)	63.73 (9.41)	52.19	2	.001	ASD > SSD > Control
				(n = 31)	(n = 18)	*U*		*p*	
ADOS-2			-	11.61 (3.54)	3.89 (3.79)	38.50		.001	SSD < ASD
ADOS-Communication			-	4.55 (1.88)	1.17 (1.38)	37.50		.001	SSD < ASD
ADOS-Social Interaction			-	7.06 (2.56)	2.67 (2.85)	72.00		.001	SSD < ASD
ADOS-Restrictive and Repetitive Behaviors			-	2.06 (1.26)	.44 (.71)	77.00		.001	SSD < ASD
					(n = 20)				
PANSS-P			-	-	10.80 (3.21)				
PANSS-N			-	-	12.85 (4.99)				
PANSS-General Psychopathology			-	-	25.55 (3.87)				
Psychopharmacological Treatment									
Antipsychotic			0.0%	0.0%	95.5%				
Antidepressant			0.0%	11.4%	18.2%				
Anxiolytic			0.0%	2.9%	50.0%				
Hypnotics			0.0%	0.0%	9.1%				
Mood stabilizer			0.0%	0.0%	13.6%				
Methylphenidate			0.0%	5.7%	0.0%				
Other psychopharmacological treatment			0.0%	8.6%	22.7%				
Education Level									
Mandatory school			3.1%	60.0%	77.3%				
University			96.9%	40.0%	22.7%				
Professional status									
Student			43.8%	42.9%	4.5%				
Employed			53.1%	14.3%	0.0%				
Unemployed			0.0%	42.9%	81.8%				
Retired			3.1%	0.0%	13.6%				

SD: Standard deviation; ADOS: Autism Diagnostic Observation Schedule; AQ-S: Autism Quotient Short; WAIS: Wechsler Adult Intelligence Scale; IQ: Intelligence Quotient; PANSS: Positive and Negative Syndrome Scale

^a^ Pair-wise group comparison. Arrows indicate direction of significant group differences, while equal signs indicate no statistically significant group difference

^b^ Bonferroni correction for multiple comparisons was used, statistical significance at *p* = .05

### Procedures

Prior to testing, informed consent was obtained from the participants and parental consent was obtained in the case of underage participants. Tests were administered individually in two or three sessions and each session had a maximum duration of 60–70 min. Sessions were conducted by an experienced researcher who was always present during each session to resolve any doubts.

### Materials

#### Wechsler Adult Intelligence Scale-IV (WAIS-IV)

We assessed the IQ of all participants with the Wechsler Adult Intelligence Scale-IV – WAIS-IV (Wechsler 2012). As an inclusion criterion for this study, individuals had to score above the cut-off point of ≥ 70 on Verbal IQ, Performance IQ, and Full-Scale IQ. While no group differences were found on Full-Scale IQ in ASD and the Control group, SSD participants showed to have a significantly lower IQ than the other two groups. It is worth mentioning that language in SSD, as assessed by verbal IQ, was relatively high, which is unusual for this condition (see Table 1).

### The Dysexecutive Questionnaire-Spanish (DEX-Sp)

The Dysexecutive Questionnaire (DEX) Spanish version (DEX-Sp) (Llanero-Luque et al., 2008; Wilson et al., 1996) is a 20-item self-report questionnaire that entails different questions related to problems in everyday life EFs (see Table 2). It has shown an adequate internal consistency and convergent validity in different language versions (Pedrero-Pérez et al., 2009; Yang et al., 2018). The test is suitable for ages between 16 and 87 and is designed to screen observable everyday manifestations of executive dysfunctions such as problems in attention, memory deficits, information processing, behavioral control, emotion regulation and others (Azouvi et al., 2015; Simblett & Bateman, 2011). Participants rate the items on a five-points Likert scale (0–4) where each point represents the severity of the problem from the perspective of the respondent, ranging from “never” to “very often.” Scores below 18 points are attributed to individuals without dysexecutive problems, scores ranging 19–28 suggest a moderate DS and scores above 28 points reflect significant impairments in day-to-day EFs (i.e., DS). Pedrero-Pérez et al., (2009) identified two scales/factors for the DEX-Sp, the Disorganization/Apathy Scale and Disinhibition/Impulsivity Scale. The first factor, Disorganization/Apathy Scale is composed of items that explore difficulties to engage or maintain a behavior as well as to organize and perform a planned behavior. The second factor, Disinhibition/Impulsivity Scale, explores difficulties to inhibit responses or unwanted behaviors when these are inappropriate to the immediate context. The DEX has been widely used recently in several clinical populations such as patients with brain damage (Simblett & Bateman, 2011), schizophrenia (Chan & Manly, 2002), substance abuse (Llanero-Luque et al., 2008), Alzheimer (Shi et al., 2017), as well as children and adults with ASD (Cederlund et al., 2010; Johnston et al., 2019).

**Table 2:** Problematic areas targeted by the DEX-Sp

Disorganization/Apathy Scale	
Item 1	Abstract thinking problem
Item 4	Planning problems
Item 6	Temporal sequencing deficits
Item 7	Lack of Insights and social awareness
Item 8	Apathy and lack of drive
Item 10	Motivation
Item 11	Shallowing of affective responses
Item 17	Knowing-doing dissociation
Item 18	Distractibility
Item 19	Poor-decision making ability
Disinhibition/Impulsivity Scale	
Item 2	Impulsivity
Item 3	Confabulation
Item 5	Euphoria
Item 9	Disinhibition
Item 12	Aggression
Item 13	Lack of concern
Item 14	Perseveration
Item 15	Restlessness-Hyperkinesis
Item 16	Inability to inhibit responses
Item 20	No concern for social rules

## Vineland Adaptive Behavior Scale, Second Edition (VABS-II)

To measure adaptive behavior in our study we administered the VABS-II. This test is designed to measure an individual’s personal, social and practical competence needed for everyday living across their lifespan (Sparrow et al., 2005). The VABS-II is suitable for ages ranging from 0 to 90 years and determines age-related typical performance in everyday situations. We used the VABS-II Survey Interview Form. In the case of adult participants, the VABS-II was administered by the interviewer directly to the adult, while in the case of underage participants, the VABS-II was administered to the participants’ parents. The VABS-II has four principal domains: Communication, Daily Living Skills, Socialization and Motor Skills. We only administered the Daily Living Skills Domain (DLS), which gathers information on individual ability to take care of themselves, accomplish household chores, or follow community rules, among other practical daily living skills (Sparrow et al., 2005). The DLS Domain is constituted by the DLS-Personal Subdomain, DLS-Domestic Subdomain and DLS-Community Subdomain. The standard score for the DSL Domain has a mean of 100 and a standard deviation of 15, whereas the scale scores for the subdomains have a mean of 15 and a standard deviation of 3.

## Results

### Everyday executive functioning analysis

Analyses were performed using IBM SPSS Statistics, version 26.0 (IBM Corp., 2019). The descriptive statistics on the DEX-Sp and its subscales can be found in Table 3. The mean total score of the ASD group in the DEX-Sp showed a DS, meaning significant impairments in everyday life aspects. Meanwhile, the SSD group showed a moderate DS and the Control group showed no impairments. The scores on the Disorganization/Apathy subscale obtained by the ASD group demonstrated a moderate DS; meanwhile, the SSD and the Control group showed no everyday EF impairments in this subscale. Lastly, the scores obtained from the three groups in the Disinhibition/Impulsivity subscale showed individuals without real-world EF difficulties.

**Table 3:** DEX-Sp and VABS-II scores

	Groups		
	Control n = 32	ASD n = 35	SSD n = 22
	Adjusted Means (SE) [95% CI]		
DEX-Sp Total Score	14.87 (2.06) [10.77–18.97]	38.05 (1.87) [34.33–41.77]	21.69 (2.84) [16.05–27.34]
Disorganization/Apathy Subscale	7.65 (1.15) [5.36–9.94]	21.16 (1.15) [19.08–23.24]	11.02 (1.59) [7.86–14.18]
Disinhibition/Impulsivity Subscale	7.22 (1.10) [5.02–9.42]	16.89 (1.00) [14.89–18.89]	10.67 (1.52) [7.64–13.70]
VABS-II-DLS Domain	90.92 (2.13) [86.68]	71.11 (1.94) [67.26–74.96]	79.29 (2.94) [73.38–85.06]
DLS-Personal	13.91 (2.61) [12.97–14.85]	11.71 (3.02) [10.68–12.75]	13.18 (2.54) [12.06–14.31]
DLS-Domestic	14.22 (2.80) [13.21–15.23]	8.83 (2.95) [7.82–9.84]	11.45 (2.50) [10.35–12.56]
DLS-Community	14.34 (1.95) [13.64–15.04]	10.11 (2.21) [9.36–10.87]	10.73 (1.61) [10.01–11.44]

DEX-Sp: Dysexecutive Syndrome Questionnaire-Spanish version; VABS-II: Vineland Adaptive Behavior Scale-II; DLS: Daily Living Skills

### IQ, age and adaptive behavior

Intellectual Quotient level and age may be relevant factors to be considered when studying adaptive functioning of individuals with ASD and SSD. To address the aspect of intellectual functioning and adaptive behavior in our study, we attempted to match the groups on IQ. However, matching on IQ was only possible for the control and ASD groups and not for the SSD group. We could not match the groups for age either, therefore, following the procedures in similar studies, we decided to control for IQ and age, in order to account for this type of variability in our analysis (see Bertollo & Yerys 2019; Martin et al., 2015).

We ran ANCOVA followed by post hoc group comparison analysis performed with Bonferroni adjustment (*p =* .05). We found a significant main effect in the DEX-Sp Total Score (*F*(2, 82) = 38.98, *p* = .001, η^2^ = 0.49) between the groups when controlling for age and IQ. The group effect size was medium. Post hoc showed significant group differences between the Control and ASD group (mean difference of 23.18, *p* = .001) and the ASD and SSD group (mean difference of 16.36, *p* = .001). No group difference was found between the Control and SSD group. Whereas, a significant main effect was found in the Disorganization/Apathy Subscale (*F*(2, 82) = 43.22, *p* = .001, η^2^ = 0.513) between the groups; the effect size was considered large. Post hoc showed a significant group difference between the Control and ASD group (mean difference of 13.51, *p* = .001) and the ASD and SSD group (mean difference of 10.14, *p* = .001); no group difference was found between the Control and SSD group. There was a significant main effect in the Disinhibition/Impulsivity Subscale (*F*(2, 82) = 22.98, *p* = .001, η^2^ = 0.359) between the groups, the effect size was medium. Post hoc tests showed significant differences between the Control and ASD group (mean difference of 9.67, *p* = .001) and the ASD and SSD group (mean difference of 6.22, *p* = .005); no significant group difference was found between the Control and SSD group.

**Fig. 1:**
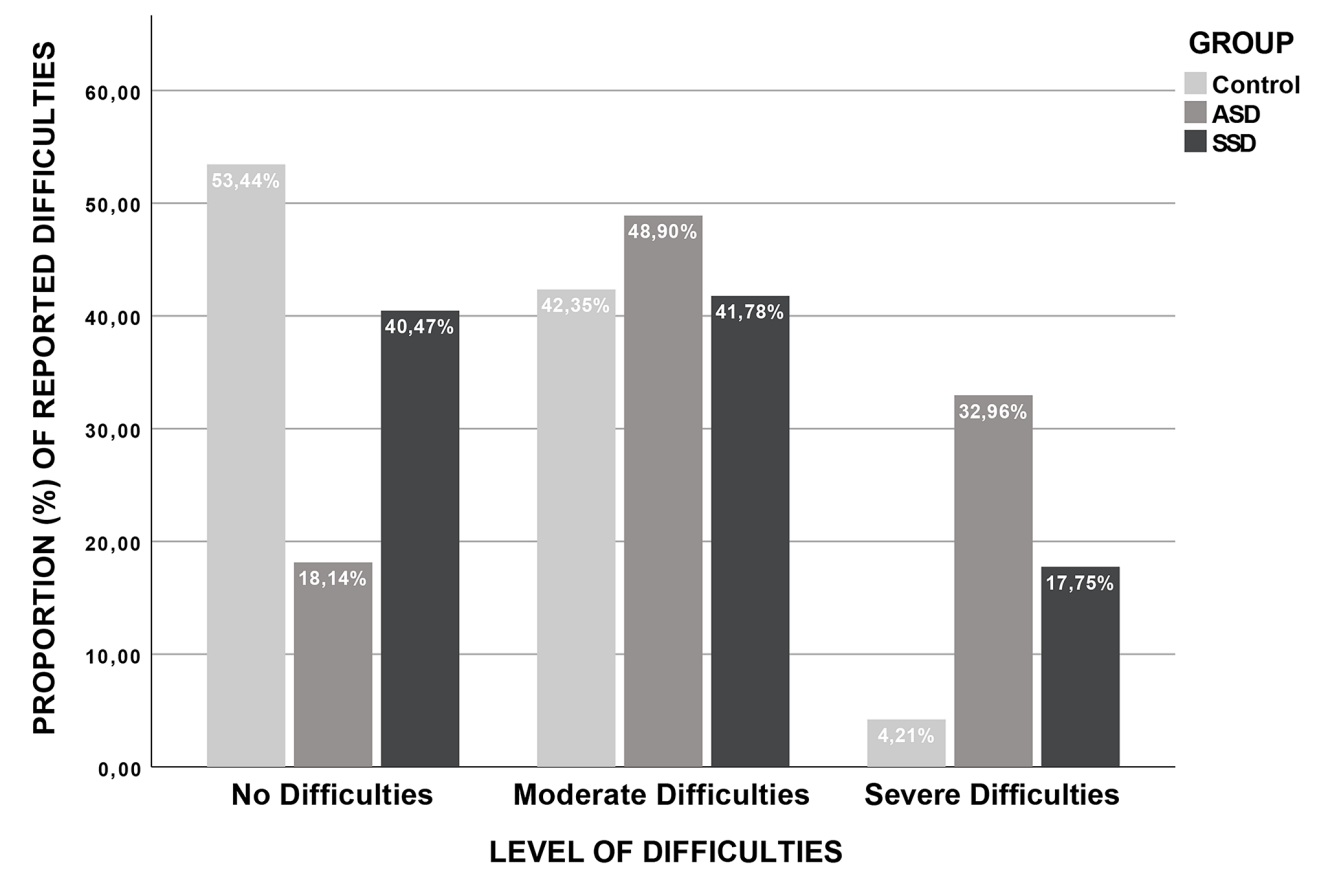
Difficulty levels reported by participants in each group

Also, we ran a Crosstab to determine the frequency of the scores obtained from the DEX-Sp regarding the difficulty levels reported (i.e., no difficulties, moderate, and severe difficulties) by each group. As seen in Fig. 1, only a few participants from the ASD group reported no difficulties, compared to the SSD group in which a greater number of participants reported no difficulties, and most participants from the Control group refer no difficulties at all. As for moderate difficulties, a greater number of participants in the ASD group reported moderate difficulties, followed by the Control group and the SSD group. Regarding severe difficulties, the ASD group reported the highest level of difficulties, followed by the SSD group and the Control group with the least severe difficulties.

### The role of everyday executive functions in adaptive behavior

Descriptive statistics obtained from the VABS-II DLS Domain can be found in Table 3. The participants in the ASD group showed overall lower performance in all three subdomains, in comparison to the participants in the SSD and Control groups. The VABS-II Scores in DLS Domain indicated a *Moderately High* adaptive level for the Control group participants, an *Adequate* adaptive level for the SSD participants and a *Moderately Low* adaptive level for the ASD group.

Furthermore, ANCOVA was run to examine the performance in the VABS-II DLS Domain, while controlling for age and IQ, followed by a post hoc group comparison analysis performed with Bonferroni adjustment (*p =* .05). We found a significant group main effect (*F*(2, 82) = 24.80, *p* = .001, *η* ^*2*^ = 0.377), with a medium effect size. Significant differences were found between the Control and ASD group (mean difference of 19.82, *p* = .001) and the Control and SSD group (mean difference of 11.70, *p* = .013), no significant group differences were found between the ASD-SSD group. The DLS subdomain scores were not suitable to run the ANCOVA since the overall model was non-normally distributed, as assessed by Shapiro-Wilk’s test (*p* < .05).

A hierarchical multiple regression was run separately for each group to determine whether everyday executive functions measured by the DEX-Sp could predict adaptive functioning in the participants using the VABS-II DLS Domain scores as the dependent variable. Following the procedures on similar studies, we also controlled for age and IQ (see Table 4). All assumptions for a multiple regression analysis were met (i.e., no outliers were detected greater than ± 3 standard deviations). For the Control group, neither the regression model including Age, WAIS-FSIQ, WAIS-VIQ, WAIS-PIQ (Model 1), nor the full model with the DEX-Sp included (Model 2) was statistically significant (*R* ^2^ = 0.226, *F*(5, 26) = 1.518, *p* = .218; Δ*R* ^2^ = 0.077). We found the same results for the SSD group for Model 1 and Model 2, (*R* ^2^ = 0.020, *F*(5, 16) = 0.066, *p* = .996; Δ*R* ^2^ = − 0.286). As for the ASD group, both models were statistically significant (*R* ^2^ = 0.440, *F*(5, 29) = 4.551, *p* = .003; Δ*R* ^2^ = 0.343), with a medium effect size. Our results showed that the impairments of EF measured by DEX-Sp did not predict adaptive behavior for the Control and SSD group. However, more severe impairments in everyday EFs predicted poorer outcomes in adaptive behavior in the participants with ASD. We also conducted our analysis without those participants from the ASD group who were under psychopharmacology treatment, and no significant differences from the ones already reported were found.

**Table 4:** Hierarchical Multiple Regression Predicting Adaptive Behavior From DEX-Sp

Adaptive Behavior – VABS-II DLS Domain												
	Control Group				ASD Group				SSD Group			
	Model 1		Model 2		Model 1		Model 2		Model 1		Model 2	
Variable	B	β	B	β	B	β	B	β	B	β	B	β
Constant	73.24^*^		68.13		32.01^*^		13.43^*^		85.51^**^		88.04^**^	
Age	− .25	− .24	− .07	− .06	.32^*^	.31^*^	.32^*^	.31^*^	− .10	− .10	− .07	− .07
WAIS-FSIQ	− .38	− .52	− .67	− .93	.39	.74	.39^*^	.74	.14	.24	.07	.12
WAIS-VIQ	.34	.47	.55	.76	.03	.06	.02	.04	− .10	− .21	− .09	− .18
WAIS-PIQ	.25	.39	.38	.59	− .15	− .28	− .14	− .28	− .05	− .10	− .03	− .05
DEX-Sp Total Score			− .52	− .31			.06	.05			− .08	− .10
*R* ^2^	.154		.226		.437		.440		.01		.02	
*F*	1.233		1.518		5.821^**^		4.551^*^		.06		.07	
Δ*R* ^2^	.154		.072		.437^**^		.003		.01		.01	
Δ*F*	1.233		2.405		5.821^**^		.140		.06		.11	

VABS-II DLS Domain: Vineland-II Adaptive Behavior Scale, Daily Living Skills Domain; WAIS: Weschler Adults Intelligence Scale; FSIQ: Full-Scale Intelligence Quotient; VIQ: Verbal Intelligence Quotient; PIQ: Performance Intelligence Quotient; DEX-Sp: Dysexecutive Syndrome Questionnaire-Spanish version. ^*^ *p* < .05, ^**^ *p* < .001

## Discussion

The current study compared everyday executive functions in a cohort of adults with ASD, SSD and controls. The novelty of our work was to examine participants’ performance in day-to-day life activities using the DEX-Sp and further explore group differences in everyday executive functioning and adaptive behaviors. In line with past research, we expected both groups to report problems in their everyday executive functioning, with more pronounced difficulties in the ASD group compared to the SSD group. Indeed, the results supported our predictions whereby individuals with ASD showed greater deficits in everyday EFs. Our data also showed a significantly lower adaptive behavior level in ASD compared to the SSD group. Significant to severe impairments were present in the ASD group and moderate impairments were detected in the SSD group. The findings of our study can potentially inform the necessary efforts that professionals should make to improve everyday life skills for these clinical populations and help the personalization of the treatments that individuals with ASD and SSD receive (Fiksinski et al., 2019; Geurts & Vissers, 2012).

The scores obtained from the DEX-Sp showed that individuals with ASD have significant deficits in everyday life aspects related to EFs, which might essentially limit their capacity to be fully independent (Pedrero-Pérez et al., 2011; Wilson et al., 1996). This outcome is similar to other studies that showed that individuals with ASD struggle on a regular basis with various aspects of daily living (Gardiner & Iarocci, 2018; Wallace et al., 2016). As stated previously, participants with ASD obtained the highest scores on the DEX-Sp, followed by the SSD group, who reported moderate impairments. Moreover, ASD participants’ scores on DEX-Sp resembled a pattern of results commonly seen in a DS. The performance of individuals with SSD was, on the other hand, associated with a moderate DS.

Overall, we can infer from the distribution of the reported difficulties on the DEX-Sp, that most of the participants in the SSD group reported moderate difficulties and only a few SSD participants expressed having severe difficulties. In ASD, we can see a different distribution of the difficulty levels reported compared to the other groups. For instance, in the ASD group, a few participants expressed having no difficulties, a larger number reported moderate difficulties and a relatively higher number of ASD participants rated their difficulties as severe. By analyzing the subscales from the DEX-Sp, the items that were grouped in the Disorganization/Apathy Subscale were significantly higher in the ASD group than the other groups. The scores obtained suggest a moderate impaired capacity for individuals with ASD, and no impairments for individuals with schizophrenia, denoting that participants with ASD would have exacerbated deficits in tasks or events that require organization and planning either to start or maintain a behavior when needed. For example, some areas that were affected in the ASD group could be abstract thinking, planning, temporal sequencing, insights and social awareness (see Table 2). However, even though only the ASD group showed impairments in these areas, it is worth noting that SSD individuals also showed higher scores than typically developed individuals. These might be considered borderline scores. We must acknowledge this finding, because they represent possible problematic areas for the participants with schizophrenia.

For those items composing the Disinhibition/Impulsivity Subscale, we found that both ASD and SSD participants reported moderate impairments, implying difficulties with inhibiting responses or inappropriate behaviors. That is, both groups showed difficulties in areas related to *impulsivity, confabulation, euphoria, disinhibition aggression, lack of concern, perseveration, restlessness-hyperkinesis, inability to inhibit responses and no concern for social rules.* We can infer, therefore, that both groups have difficulties generating appropriate goal-directed behaviors.

Literature in schizophrenia suggests that older individuals have more severe EF impairments than individuals in early stages of the disorder (Muralidharan et al., 2020). That is to say that worsening deficits in executive functions are seen in older adults with schizophrenia compared to middle-aged and younger individuals who have fewer difficulties to overcome everyday problems related to EFs (Martin et al., 2015). These findings were not supported by our results. The participants in the SSD group were significantly older than the ASD participants, yet they showed less severe impairments in everyday EFs compared to the ASD group.

Executive functions have been shown to be a robust predictor of lower adaptive behavior in ASD (Kenworthy et al., 2008; Matthews et al., 2015; Wallace et al., 2016) as well as in SSD (Bhattacharya, 2015; Fiksinski et al., 2019; Marinopoulou et al., 2016). Therefore, we anticipated that higher scores in the DEX-Sp (i.e., greater impairment) would predict lower scores in the VABS-II DLS Domain (i.e., greater problems in adaptive behaviors). This was indeed the case, but only in the ASD group, which reported severe difficulties. Their difficulties were related to generating appropriate behaviors, elaborating strategies, and to self-organization needed to initiate goal-directed behaviors. Subsequently, it seems that the DEX-Sp might have a predictive value for severe but not moderate deficits in EF. Our findings are in line with past autism research which suggest that EF plays a more important role than intellectual functioning in adaptive behaviors (Gardiner & Iarocci 2018; Tillmann et al., 2019). We found that real-world EFs (measured by DEX-Sp) were a stronger predictor for adaptive behavior (measured by VABS-II) than age and IQ. As for the SSD group, IQ, age and everyday-EFs did not predict adaptive behavior. This suggests that other variables, such as schizophrenia core symptoms, may be intervening with the deficits seen in daily living skills.

In summary, based on our findings, the DEX-Sp has shown to be adequate for assessing everyday difficulties related to executive functioning, as it has demonstrated to trace well the similarities and differences between EF cognitive abilities in ASD and SSD populations. We believe that our study supports the use of DEX-Sp in clinical settings as an assessment tool for everyday EFs and suggest that it could be a useful tool to evaluate the efficiency of interventions directed at adults with ASD and SSD. Having a screening tool that identifies and evaluates accurately EF abilities could be of great importance at a clinical level. The easy administration and the wide range of areas that DEX-Sp covers, provides a clear insight into how individuals in these clinical populations perceive their difficulties and how these might affect their independence. For instance, because individuals with ASD show more EF problems, it is likely that they might need a greater number of sessions than individuals with SSD. Also, given that adults with ASD showed more difficulties on the Disorganization/Apathy Subscale of DEX-Sp, their intervention should be more focused on skills such as organization, planning and behavior initiation. As for SSD, interventions would need to focus primarily on the problematic characteristics related to the Disinhibition/Impulsivity Subscale, with a focus on promoting appropriate goal-directed behaviors. Thus, in general, our study provides a better characterization and differentiation of problematic EF areas and, consequently, might help to improve individuals’ everyday life functioning. Undoubtedly, more research is still necessary to establish the usefulness of the DEX-Sp as an evaluation tool to assess the effectiveness of EF programs or interventions in adults with autism and schizophrenia in daily life contexts.

## Limitations

Our study has some limitations that need to be accounted for in future research. For example, our data on SSD should be treated with caution given its small sample size. Also, it is important to mention that most of our SSD participants have been part of integration to the community program of the Psychiatry Unit of the Hospital during a prolonged time. Therefore, they have received continued treatment and interventions at the time of the study. Also, because the rate of responsiveness to our study was low with younger patients with SSD, further research would need to include younger participants with schizophrenia. The role of antipsychotic medication should also be analyzed in future studies, as medication may intervene on the individuals’ ability to cope in everyday settings.

Also, we used the DEX-Sp self-report version in our study and we think that it would be useful to compare participants’ reports with the informant-report version of the DEX. Unfortunately, this version is not available in Spanish. Informant-report would ascertain whether our result could have been affected by the lack of deficit awareness in the clinical groups (Simblett & Bateman, 2011). It is worth mentioning, however, that in the English version of DEX, no differences between perceived EF difficulties were found between adults with ASD and the parent-caregivers (Hill & Bird, 2006).

## Conclusions

This study found greater deficits in everyday executive functions associated with ASD and with a poor adaptive behavior. Our results showed that adults with ASD in this study reported severe difficulties in their abilities to initiate or maintain a behavior with the purpose of organizing or planning activities effectively. We also found shared executive function deficits within ASD and SSD groups compared to typically developing controls in areas related to inhibiting inappropriate behavioral responses or generated impulsive behaviors. The SSD group denoted moderate impairments in EFs, however we did not find an association between their scores on the DEX-Sp and the scores obtained on the VABS-II. Our findings add evidence to existing literature in children and adolescents with ASD, indicating that deficits in everyday executive functioning continue into adulthood. As for adults with SSD, moderate difficulties on EF seem to remain relatively constant throughout development.

## Data Availability

The datasets used and/or analyzed during the current study are available from the corresponding author on reasonable request.
