# Correction to: Differences in Daily Life Executive Functioning Between People with Autism and People with Schizophrenia

**DOI:** 10.1007/s10803-022-05613-z

**Published:** 2022-05-18

**Authors:** Jo A. Yon-Hernández, Dominika Z. Wojcik, Laura García-García, Manuel A. Franco-Martín, Ricardo Canal-Bedia

**Affiliations:** 1 Instituto Universitario de Integración en La Comunidad (INICO), Universidad de Salamanca, Avda. de la Merced, 109-131, 37005 Salamanca, Spain; 2 Zamora Hospital (Complejo Asistencial de Zamora), Zamora, Spain

## Correction to: Journal of Autism and Developmental Disorders 10.1007/s10803-022-05547-6

During production of this article there was an error in the display of the Fig. 1.

The original article has been revised (Fig. 1).

**Fig. 1:**
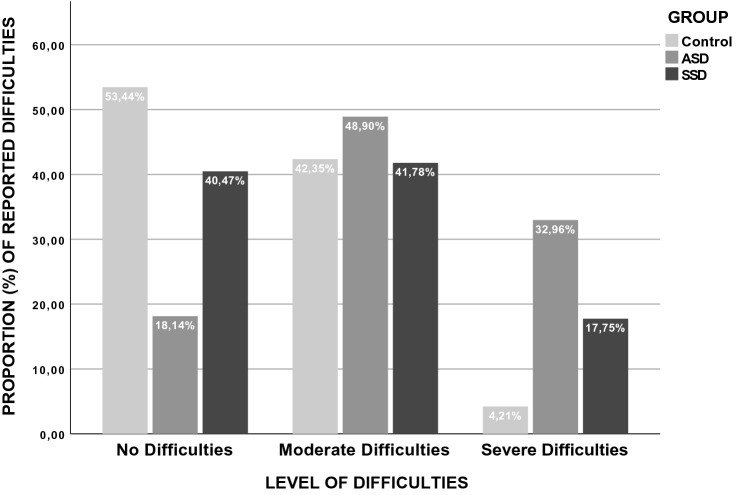
Difficulty levels reported by participants in each group

